# Topography of calcium phosphate ceramics regulates primary cilia length and TGF receptor recruitment associated with osteogenesis

**DOI:** 10.1016/j.actbio.2017.04.004

**Published:** 2017-07-15

**Authors:** Jingwei Zhang, Melis T. Dalbay, Xiaoman Luo, Erik Vrij, Davide Barbieri, Lorenzo Moroni, Joost D. de Bruijn, Clemens A. van Blitterswijk, J. Paul Chapple, Martin M. Knight, Huipin Yuan

**Affiliations:** a Complex Tissue Regeneration Department, MERLN Institute for Technology Inspired Regenerative Medicine, Maastricht University, Universiteitsingel 40, 6229 ER, The Netherlands; b Institute for Translational Medicine, Zhejiang University, Hangzhou, Zhejiang Province 310029, China; c Institute of Bioengineering and School of Engineering and Materials Science, Queen Mary University of London, Mile End Rd, London E1 4NS, UK; d Xpand Biotechnology BV, Bilthoven, The Netherlands; e Department of Biomaterials Science and Technology, MIRA Institute for Biomedical Technology and Technical Medicine, University of Twente, P.O. Box 217, 7500AE Enschede, The Netherlands; f William Harvey Research Institute, Queen Mary University of London, Charterhouse Square, London E1 4NS, UK; g National Engineering Research Center for Biomaterials, Sichuan University, 610064 Chengdu, China

**Keywords:** Calcium phosphate ceramic, Topography, Mesenchymal stromal cell, Primary cilia, TGFβ, Bone

## Abstract

The surface topography of synthetic biomaterials is known to play a role in material-driven osteogenesis. Recent studies show that TGFβ signalling also initiates osteogenic differentiation. TGFβ signalling requires the recruitment of TGFβ receptors (TGFβR) to the primary cilia. In this study, we hypothesize that the surface topography of calcium phosphate ceramics regulates stem cell morphology, primary cilia structure and TGFβR recruitment to the cilium associated with osteogenic differentiation. We developed a 2D system using two types of tricalcium phosphate (TCP) ceramic discs with identical chemistry. One sample had a surface topography at micron-scale (TCP-B, with a bigger surface structure dimension) whilst the other had a surface topography at submicron scale (TCP-S, with a smaller surface structure dimension). In the absence of osteogenic differentiation factors, human bone marrow stromal cells (hBMSCs) were more spread on TCP-S than on TCP-B with alterations in actin organization and increased primary cilia prevalence and length. The cilia elongation on TCP-S was similar to that observed on glass in the presence of osteogenic media and was followed by recruitment of transforming growth factor-β RII (p-TGFβ RII) to the cilia axoneme. This was associated with enhanced osteogenic differentiation of hBMSCs on TCP-S, as shown by alkaline phosphatase activity and gene expression for key osteogenic markers in the absence of additional osteogenic growth factors. Similarly, *in vivo* after a 12-week intramuscular implantation in dogs, TCP-S induced bone formation while TCP-B did not. It is most likely that the surface topography of calcium phosphate ceramics regulates primary cilia length and ciliary recruitment of p-TGFβ RII associated with osteogenesis and bone formation. This bioengineering control of osteogenesis via primary cilia modulation may represent a new type of biomaterial-based ciliotherapy for orthopedic, dental and maxillofacial surgery applications.

**Statement of Significance:**

The surface topography of synthetic biomaterials plays important roles in material-driven osteogenesis. The data presented herein have shown that the surface topography of calcium phosphate ceramics regulates mesenchymal stromal cells (e.g., human bone marrow mesenchymal stromal cells, hBMSCs) with respect to morphology, primary cilia structure and TGFβR recruitment to the cilium associated with osteogenic differentiation *in vitro*. Together with bone formation *in vivo*, our results suggested a new type of biomaterial-based ciliotherapy for orthopedic, dental and maxillofacial surgery by the bioengineering control of osteogenesis via primary cilia modulation.

## Graphical abstract

**Figure:**
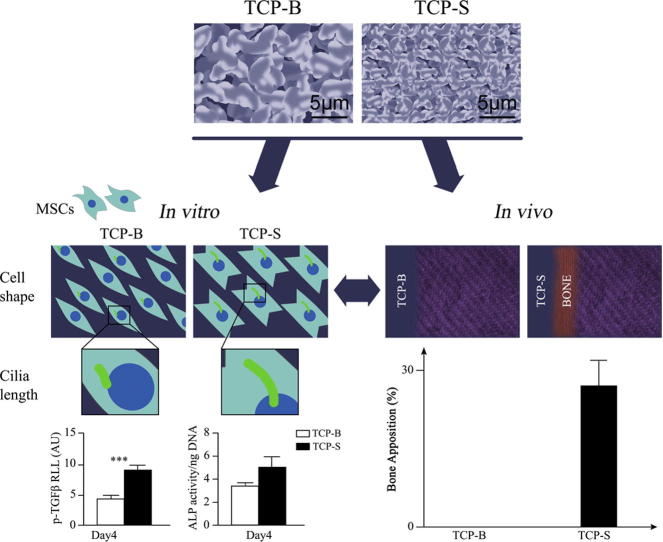

## 1 Introduction

Calcium phosphate (CaP) ceramics are widely used in orthopedic, dental and maxillofacial surgery as bone substitutes because of their chemical homology to native bone mineral, excellent biocompatibility and the ability to support osteogenesis on their surface (i.e. osteoconductivity) [1], [2], [3]. However, osteoinductivity of bone graft substitutes, i.e. the ability to positively induce osteogenic differentiation of stem cells to form bone, is required for bone regeneration in critical-sized bone defects [4], [5]. The most common approach to make CaP ceramics osteoinductive is to combine them with growth factors (e.g. bone morphogenetic proteins, BMPs) [6]. However, the cost and safety of such approaches pose major concerns [7].

In the last decades, a subclass of CaP ceramics has been engineered to impart osteoinductivity without adding any osteogenic component, but only by tailoring their physico-chemical properties [8]. Among physico-chemical properties important for osteoinductivity, micropores (i.e. pores smaller than 10 µm) have long been recognized as a crucial material factor. Given the same chemistry, macroporous hydroxyapatite (HA) ceramics with micropores on their surface gave rise to bone formation following either subcutaneous [9] or intramuscular [10] implantation, while those without micropores failed. Similarly for CaP ceramics, the osteoinductive potential was also found to be correlated with microporosity such that higher microporosity resulted in greater osteoinductivity [11]. It has recently been shown that when two tricalcium phosphate (TCP) ceramics, having the same chemistry and microporosity were intramuscularly implanted, the one presenting submicron-scaled pores (0.65 ± 0.25 µm, TCP-S) induced ectopic bone formation while the other with micro-scaled pores (1.58 ± 0.65 µm, TCP-B) did not [12], [13]. The present study aimed to further examine the influence of surface topography on osteogenesis.

Despite several studies showing the influence of surface topography on cell differentiation both in 2D and in 3D cell culture systems [13], [14], the biological mechanism with which stem cells respond to surface structures and undergo osteogenic differentiation remains unclear. Previous findings have indicated that substrate topography regulates cell morphology to control differentiation into specific lineages [14], [15]. For example, Guvendiren and Burdick showed that the size and pattern of surface wrinkles influenced MSCs morphology thereby regulating differentiation [16]. It has also been shown that cell morphology regulates MSC differentiation through mediation of RhoA activity without the requirement of soluble factors [17].

Primary cilia are single microtubule based hair like structures that respond to chemical and mechanical changes in the extracellular environment, coordinating multiple signalling pathways such as receptor tyrosine kinase (RTK), Hedgehog (Hh), Wnt, Notch, mTOR and mechanotransduction [18], [19]. It has been previously shown that primary cilia respond to changes in surface topography with MSC cilia elongation on grooved topographies [20]. This cilia response was mediated by changes in cell and actin morphology and was shown to regulate Wnt signalling. Furthermore primary cilia are required for osteogenic differentiation of MSCs [21] but how this regulation occurs is not known. It has been shown that recruitment of TGFR to the primary cilium is necessary for downstream TGF signalling [22] which is an important regulator of osteogenesis [23]. We therefore hypothesized that changes in surface topography of CaP ceramics may regulate cell morphology, primary cilia expression and ciliary recruitment of TGFR associated with osteogenesis.

To test this hypothesis, we investigated the morphology and primary cilia expression of hBMSCs on TCP ceramics with two distinct surface topographies (namely TCP-B and TCP-S). We investigated cell morphology, primary cilia expression, TGFR recruitment to the cilium and osteogenic differentiation *in vitro* and bone formation in an *in vivo* canine ectopic model. Here we show for the first time that hBMSCs grown on CaP ceramics with submicron surface topographies undergo osteogenic differentiation associated with changes in primary cilia structure and increased ciliary p-TGFβRII.

## 2 Materials and methods

### 2.1 Preparation of TCP-S and TCP-B ceramics

TCP powders were prepared as previously described [13]. Briefly, a calcium hydroxide (Fluka) suspension and a phosphoric acid (Fluka) solution were mixed at a Ca/P ratio of 1.50. TCP-S and TCP-B powders were obtained by controlling the respective reaction rates. The green bodies were then obtained after mixing the TCP-S and TCP-B powders with diluted H_2_O_2_ (0.1%) (Merck). The TCP-S and TCP-B ceramics were finally achieved by sintering the dry green bodies at 1050 °C (TCP-S) and 1100 °C (TCP-B) for 8 h, respectively.

**Fig. 1:**
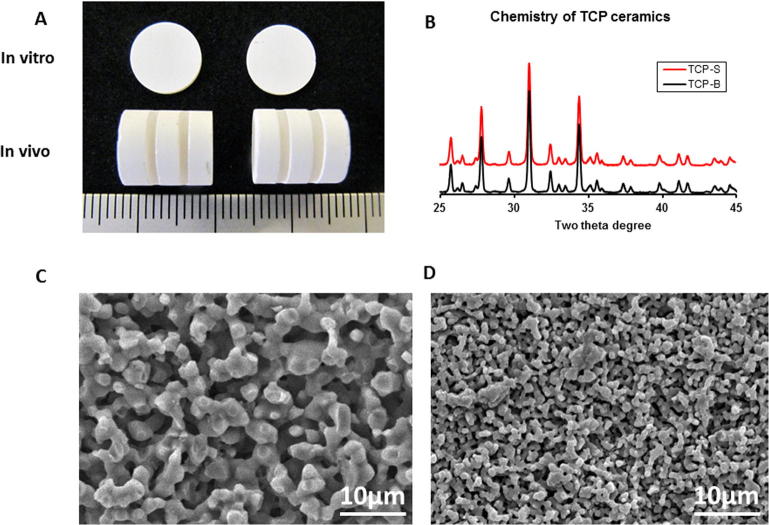
TCP ceramics were created with identical chemistry but different surface topography as shown by XRD and SEM respectively. Images of samples used for *in vitro* and *in vivo* evaluations (A); chemistry of TCP ceramics analyzed with XRD (B); SEM images of TCP-B (C) and TCP-S (D).

TCP-S and TCP-B discs (Φ9 × 1 mm) were machined using a diamond-coated saw microtome (SP-1600, Leica, Germany) for *in vitro* evaluation. Ceramic cylinders (Φ9 × 12 mm) with two transverse cuts of 1.1 ± 0.1 mm were made as well for *in vivo* evaluation (Fig.1A). The obtained materials were then ultrasonically cleaned with acetone, 70% ethanol and demineralized water, and dried at 80 °C. All samples were steam sterilized at 121 °C for 30 min and dried at 80 °C afterwards.

Crystal chemistry of the TCP-S and TCP-B ceramics were determined with X-ray diffraction (XRD, Rigaku, Japan) and confirmed to be β-TCP. Surface morphology was observed with an environmental scanning electron microscope (ESEM; XL30, ESEMFEG, Philips, Eindhoven, The Netherlands) in the secondary electron mode; at the same time, grain size and pore size were measured with 10 images at the magnification of 5000. Porosity, pore distribution and total pore area were determined by mercury intrusion testing (Micromeritics, USA).

### 2.2 *In vitro* cell culture

#### 2.2.1 Isolation and expansion of hBMSCs

hBMSCs from three donors were isolated from bone marrow aspirates with as previously described [13], [24], [25]. In brief, aspirates from the donors were re-suspended using a 20 G needle, plated at a density of 5 × 10^5^ cells/cm^2^ and cultured in proliferation media (PM) for expansion. PM consisted of basic media (BM) and basic fibroblasts growth factor (bFGF, Instruchemie, the Netherlands, 1 ng/mL). BM was consisted of alpha-MEM (Life Techonologies) supplemented with 10% of fetal bovine serum (FBS, Life Technologies), 0.2 mM ascorbic acid (ASAP, Life Technologies), 20 mM l-glutamine (Life Technologies), 100 U/mL penicillin (Life Technologies) and 100 µg/mL streptomycin (Life Technologies). Cells were grown at 37 °C in a humid atmosphere with 5% CO_2_, media was refreshed twice per week and cells were sub-cultured when they reached 80–90% confluency. Passage 2-3 hBMSCs were used.

#### 2.2.2 Cell culture on TCP ceramics

To study the effect of surface topography on cellular behavior, hBMSCs were cultured on the TCP discs. All the discs were placed in non-treated 48-well plate and soaked in BM for at least four hours before cell seeding. To evaluate cell morphology (actin staining) and primary cilia expression, cells were seeded onto the TCP discs at a density of 5000 cells/cm^2^. For cell attachment, SEM analysis of morphology, cell proliferation, osteogenic differentiation, gene expression and analysis of ciliary p-TGFβR II, cells were seeded at a density of 25,000 cells/cm^2^ in 1 mL basal media (BM). Additional studies were conducted in the presence of osteogenic media (OM) containing 10^−8^ M dexamethasone in addition to BM composition for gene expression. Cells were cultured on ceramic discs at 37 °C in a humid atmosphere with 5% CO_2_. The media was refreshed twice per week.

#### 2.2.3 SEM analysis of cell attachment and morphology

For cell attachment and morphology observation, cells on TCP discs were viewed at day 1 with methylene blue staining and SEM observations. After fixing with 4% paraformaldehyde and washing with PBS, the samples were stained with 1% methylene blue and viewed with a steromicroscope (LM; E600, Nikon SMZ-10A, Japan). Thereafter, the samples were dehydrated in sequential ethanol series and followed by critical point drying from liquid carbon dioxide using a Balzers CPD 030 Critical Point Dryer. The samples were gold sputter coated (Cressington) before being imaged by SEM.

#### 2.2.4 Actin staining and cell morphology analysis

To analyze cell morphology individually, cells (5000 cells/cm^2^, n = 3 per condition) were cultured on TCP ceramics for day 1 and day 4 and then fixated for 30 min in 4% paraformaldehyde, washed with PBS and permeabilised in 0.25% Triton-X 100 in PBS. Subsequently, F-actin was stained using phalloidin-AF488 (LifeTechnologies) and nuclei were stained using DAPI (Sigma Aldrich). After washing with PBS, montage images were captured using a BD Pathway system (BD Pathway 435, BD biosciences). The image analysis program Cell Profiler was used to quantitatively measure the morphological characteristics of cells cultured on TCP-B and TCP-S surface. Depicted descriptors for cellular morphology were chosen based on relevance and statistically significant differences between TCP-S and TCP-B. Such measures included the cell area and the form factor (ratio of minor over major axis lengths).

#### 2.2.5 Confocal microscopy analysis of primary cilia expression and TGFR localisation

To evaluate the effect of surface topography on primary cilia occurrence and length, cells (5000 cells/cm^2^, n = 3 per condition) cultured on TCP ceramics up to 7 days underwent serum starvation for 24 h before fixation. Cells were fixed with 4% paraformaldehyde at 37 °C for 10 min, permeabilised in 0.5% Tiriton and blocked with 5% goat serum. Primary cilia were labelled using anti-acetylated α-tubulin antibody (clone 6-11 B-1, 1:2000; Sigma-Aldrich) and pericentrin antibody (Abcam, ab448-100) at 4 °C for overnight, washed thereafter and incubated for 1 h at 25 °C with Alexa 488 anti-mouse conjugate and Alexa Fluor 594 F(ab′)2 fragment of goat anti-rabbit IgG (H+L) (Invitrogen). p-TGFβR II was labelled using p-TGFβR II (Tyr-424) (Santa Cruz Biotechnology), Sc-17007-R and Alexa Fluor 633 goat anti-rabbit IgG (H+L) (Invitrogen). Finally, the samples were mounted with a DAPI counterstaining (Invitrogen). Maximal projections of confocal z-stacks were created with a Leica SP2 confocal microscope (pixel size = 0.1 μm). Cilia prevalence was assessed based on the percentage of ciliated cells per field of view (n = 5 fields), cilia length and ciliary p-TGFR II intensity were measured using image J software as previously described [20], [26].

Additional studies were conducted to determine the effect of OM on primary cilia expression and length. For these studies, hBMSCs were cultured on glass coverslips (Ø13 mm) in 24-well plates in either BM or OM. Cells were seeded at a density of 5000 cells/cm^2^ with media refreshed at day 4. Cells were serum starved for 24 h prior to fixation at day 7 for analysis of primary cilia expression.

#### 2.2.6 Cell proliferation and ALP assays

For cell proliferation and ALP assays, samples (3 donors, 25,000 cells/cm^2^, n = 3 per condition) were harvested at day 1, day 4, day 7 and day 14 respectively. The samples were gently rinsed three times with PBS, dried by aspirating PBS, and stored at −20 °C until further use. 500 µL of lysis buffer (prepared according to manufacturer’s instructions of CyQuant Cell Proliferation Assay kit instructions) was added onto each sample, followed by three cycles of freezing and thawing at −20 °C and room temperature, respectively. Cell proliferation was analyzed with a DNA assay (CyQuant Cell Proliferation Assay kit, Sigma, the Netherlands), according to the manufacturer’s instruction. Briefly, 100 µL cell lysate and DNA standard were incubated with 100 µL CyQuant GR dye at room temperature for 15 min and measured using a spectrophotometer (Victor, Perkin Elmer) at an excitation wavelength of 480 nm and emission wavelength of 520 nm. ALP activity was measured using a CDP-star assay kit (Roche). 40 µL of CDP star substrate were incubated with 10 µL cell lysate for 20 min, after which the luminescence was measured using a spectrophotometer (Victor, Perkin Elmer). ALP expression was normalized to DNA content.

To examine the osteogenic differentiation of cells on TCP discs, ALP staining was conducted on the 7-day samples, following the manufacturer’s protocol of the Alkaline Phosphatase kit (Sigma-Aldrich). Samples were first washed three times with PBS, fixed with 4% paraformaldehyde for 30 s, and then incubated for 30 min in staining solution containing Naphtol AS MX phosphate and Fast Blue. The ALP positive cells were stained blue. Finally, the samples were washed 3 times with deionized water and observed under the stereomicroscope (LM; E600, Nikon SMZ-10A, Japan).

#### 2.2.7 PCR analysis of osteogenic gene expression

For gene expression, cells were cultured on TCP discs in both BM and OM. Samples (n = 3 per condition) were collected at day 4, day 7 and day 14. Bone-related gene expression was evaluated with quantitative real-time polymerase chain reaction (PCR) assay. RNA isolation was performed using Trizol reagent (Invitrogen) and Nucleospin RNA isolation kit (Macherey-Nagel Gmbh & Co.) according to the manufacturer’s instructions. Total RNA was measured using a NanoDrop spectrophotometer (Nanodrop technologies, USA). The RNA was used to synthesize complementary DNA (cDNA) with an iScript cDNA Synthesis kit (BioRad) according to the manufacturer’s instructions. PCR analysis was performed with a Bio-Rad real-time PCR system (Bio-Rad, Hercules, CA, USA) on alkaline phosphate (ALP), collagen type I (Col I), osteocalcin (OCN), and osteopontin (OPN), with beta-2 microglobulin (B2M) as the house-keeping gene used for normalization. Primer sequences for ALP, Col I, OCN, OPN, and B2M are listed in Table 1. The relative amounts of target genes normalized by B2M were calculated by 2^−ΔCT^ method where ΔC_T_ = C_T,Target_ − C_T,B2M_.

**Table 1:** qPCR primer sequences.

Gene	Forward primer	Reverse primer
OCN	GGCAGCGAGGTAGTGAAGAG	GATGTGGTCAGCCAACTCGT
OPN	CCAAGTAAGTCCAACGAAAG	GGTGATGTCCTCGTCTGTA
ALP	ACAAGCACTCCCACTTCATC	TTCAGCTCGTACTGCATGTC
COL-I	AGGGCCAAGACGAAGACATC	AGATCACGTCATCGCACAACA
B2M	GACTTGTCTTTCAGCAAGGA	ACAAAGTCACATGGTTCACA

### 2.3 *In vivo* bone formation assay

TCP-B and TCP-S samples were implanted in an ectopic canine model for 12 weeks. Following the permission of the local animal care committee (Animal Center, Sichuan University, Chengdu, China), the TCP-S and TCP-B cylinders (Fig.1A) were implanted in the *para*-spinal muscles of 8 adult male dogs (mongrel, 10–15 kg). All surgeries were conducted under general anaesthesia by abdominal injection of sodium pentobarbital (30 mg/kg body weight) and sterile condition. Following the surgeries, buprenorphine (0.1 mg per animal) was intramuscularly given to the animals as pain relief for 2 days, while penicillin (40 mg/kg) was intramuscularly injected for 3 consecutive days to prevent infection. After operation, the animals were allowed for full weight bearing and received normal diet. After 12 weeks, the dogs were sacrificed by a celiac injection of excessive amount of pentobarbital sodium. Implants were harvested with surrounding tissues and fixed in 4% formaldehyde, and embedded in poly (methyl methacrylate) (PMMA) after a series of gradient ethanol dehydration. Non-decalcified sections were prepared trans-crossing the transverse cuts using a diamond saw (SP-1600, Leica, Germany) and stained with 1% methylene blue (Sigma) and 0.3% basic fuchsine (Sigma) solutions. Histological observations were performed using light microscopy to evaluate bone formation in the explants. The histological slide crossing the middle of each explant was scanned with a scanner (DIMAGE Scan Elite5400 II, model AF5400-2, KONICA MINOLTA). Inner surface of the transverse cuts and the inner surface covered by bone were measured in length with the printouts of the scanned images. Bone formation was quantified as coverage of the inner surface (%).

### 2.4 Statistical analysis

Quantitative results are shown as average ± standard deviation. Multiple comparisons were performed with two-way analysis of variance (ANOVA) followed by Bonferroni post-test comparisons. P-values lower than 0.05 were considered as statistically significant differences.

## 3 Results

### 3.1 Characterization of TCP-S and TCP-B ceramics

Fig.1A shows the images of TCP samples for both *in vitro* and *in vivo* evaluation. XRD analysis revealed that the two TCP ceramics had the same chemistry of β-TCP (Fig.1B). TCP with different sizes of surface microstructure was prepared shown by SEM; TCP-B contained larger grains and micropore size than those of TCP-S leading to differences in surface topography (Fig.1C, D). Grain size, pore size, porosity, and total pore surface area were summarized in Table 2. The unique grains and pores in TCP-S and TCP-B resulted in different surface roughness in the two materials, with the Ra of 0.126 ± 0.003 µm for TCP-S and 1.287 ± 0.011 µm for TCP-B as reported in our previous study [12].

**Table 2:** Physical properties of TCP ceramics.

Physical parameters	TCP-B	TCP-S
Average grain size(μm)	3.40 ± 0.82	0.9 ± 0.25
Average pore size(μm)	1.70 ± 0.63	0.67 ± 0.30
Porosity (%)	47	45
Total pore area (m^2^/g)	0.7	1.4

### 3.2 hBMSC morphology and attachment is regulated by TCP surface topography

**Fig. 2:**
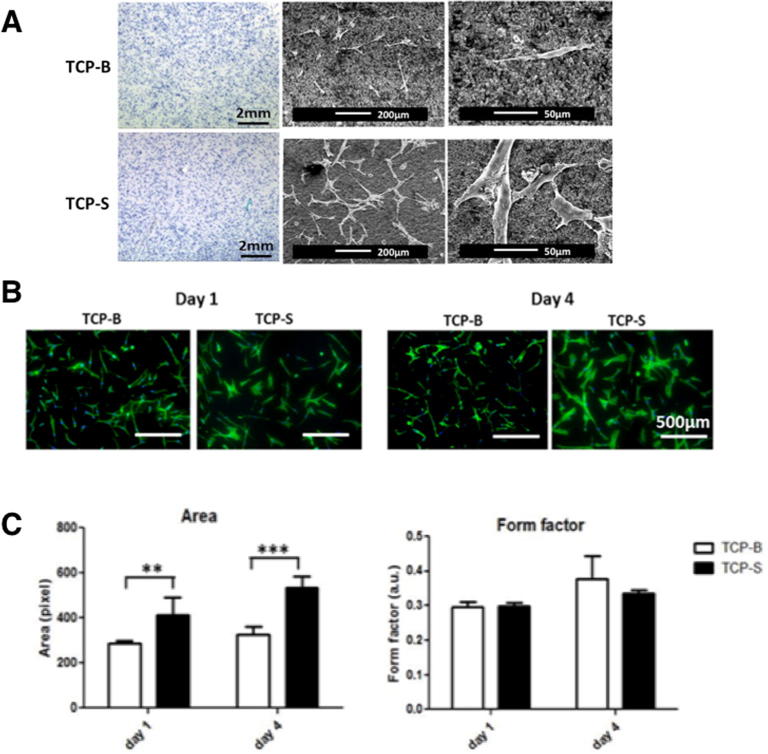
The morphology and actin organization of hBMSCs are regulated by the surface topography of TCP ceramics. A: Methylene blue staining and SEM observation of hBMSCs cultured on TCP-B and TCP-S for 24 h; B: Representative images of cell nucleus (DAPI) and actin skeleton (phalloidin) of hBMSCs on TCP discs for 1 and 4 days; C: The area and form factor plot of hBMSCs cultured on TCP-S and TCP-B for 1 and 4 days.

Cells were found to be homogeneously distributed on both TCP discs after 24 h (Fig.2A, methylene blue staining images). SEM images showed clear attachment of hBMSCs to the TCP substrate and confirmed that cells were larger and more spread on TCP-S than on TCP-B (Fig.2A). Fluorescent imaging of F-actin showed that cells cultured on TCP-S were more spread and had larger area compared to those on TCP-B after 1 and 4 days (Fig.2B). The morphological change was similar at early time on TCP-S and TCP-B between the low cell seeding density (5000 cells/cm^2^, Fig.2B) and the high cell seeding density (25,000 cells/cm^2^, Fig.2A). The quantitative data confirmed that the cells on TCP-S had significantly larger area, but similar form factor, compared to those on TCP-B (Fig.2C). The form factor values of cells ranged between 0.3 and 0.4 for both TCP ceramics, suggesting that cells had elongated morphology on both materials. However, those on TCP-S were more spread than on TCP-B, as shown by all the other morphological indicators, and had a greater level of F-actin staining. Evidently, the size of the surface microstructure influenced the morphology and cytoskeletal organization of hBMSCs cultured on TCP discs for 1 and 4 days.

### 3.3 Primary cilia elongation occurs in response to TCP-S topography and osteogenic media

**Fig. 3:**
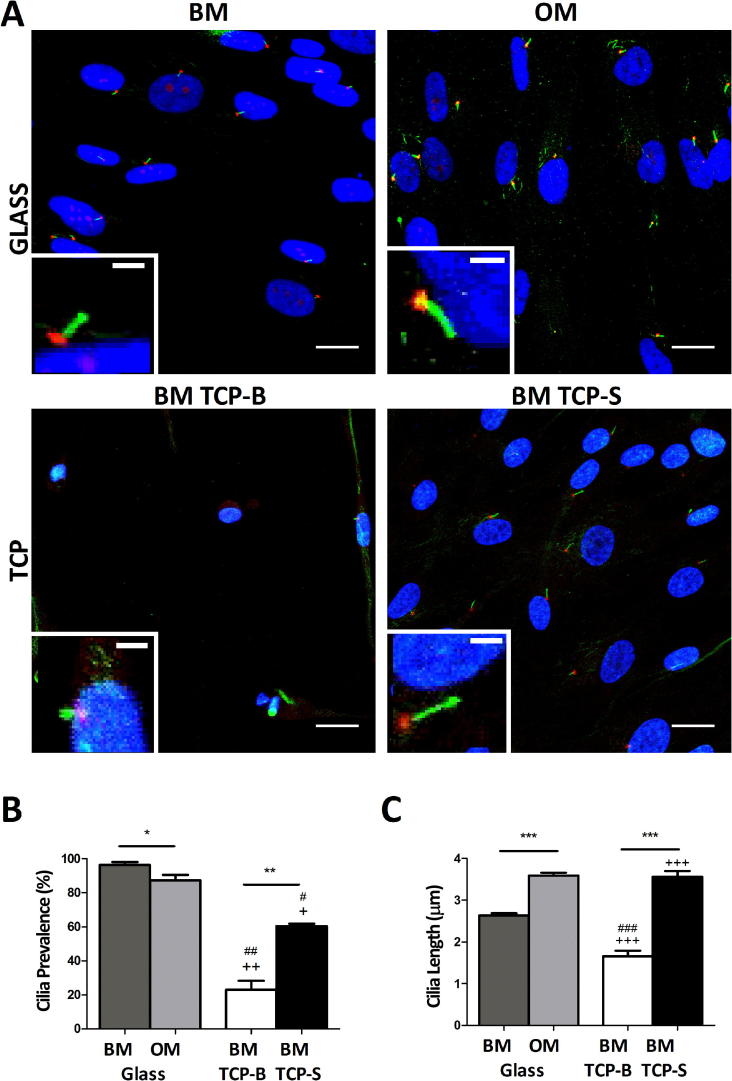
Primary cilia expression and length are modulated by osteogenic differentiation media and TCP topography. Confocal images showing primary cilia labelled with acetylated α-tubulin (green) in hBMSCs cultured on glass in either BM or OM and on TCP ceramic surfaces in BM for 7 days (A). Scale bars are 10 μm for field images and 3 μm for boxed images of single cilium. Cilia basal bodies are labelled with pericentrin (red) and nuclei with DAPI (blue). Corresponding primary cilia prevalence (n = 10 fields of view) (B) and length (n > 100 cilia) (C) (^*^p < 0.05, ^**^p < 0.001, ^***^p < 0.0001 for OM vs. BM and for TCP-S vs. TCP-B; ^+^p < 0.05, ^++^p < 0.001, ^+++^p < 0.001 for TCP vs. BM; ^#^p < 0.05, ^##^p < 0.001, ^###^p < 0.0001 for TCP vs. OM), Mann-Whitney test. (For interpretation of the references to colour in this figure legend, the reader is referred to the web version of this article.)

The α-tubulin staining indicated that the cell morphology on TCP discs and glass slice was different (Fig.3A). On TCP-S the cells were bigger in size than on TCP-B, while the cells on glass slice were more flattened as compared on TCP-S. As shown in Fig.3A and 3B primary cilia expressed by hBMSCs cultured for 7 days on both glass coverslips and TCP ceramic surfaces were fluorescently labelled for acetylated alpha tubulin and pericentrin and imaged using confocal microscopy (Fig.3A). On glass coverslips, approximately 90–100% of hBMSCs expressed primary cilia although this incidence was slightly reduced by the addition of dexamethasone present in the OM (Fig.3B) (p < 0.05). However osteogenic media significantly increased the length of primary cilia from a mean value of approximately 2.6 μm to 3.5 μm (Fig.3C, p < 0.001). Fewer primary cilia were present in cells cultured on TCP ceramics compared to glass in either culture media, with the prevalence on TCP-B being particularly low, possibly due to the fact that these cells appeared less confluent (Fig.3A and B). Both primary cilia prevalence and length were affected by TCP ceramic surfaces (TCP-S vs TCP-B). In particular, culturing cells on TCP-S induced a significant increase in cilia prevalence and length (Fig.3B, p < 0.01), compared to that observed on TCP-B (Fig.3C, p < 0.001). Primary cilia of cells cultured on TCP-S in BM were equivalent in length to those expressed by cells cultured on glass in osteogenic media with dexamethasone.

### 3.4 Primary cilia recruitment of p-TGFβ RII is increased on TCP-S surfaces

**Fig. 4:**
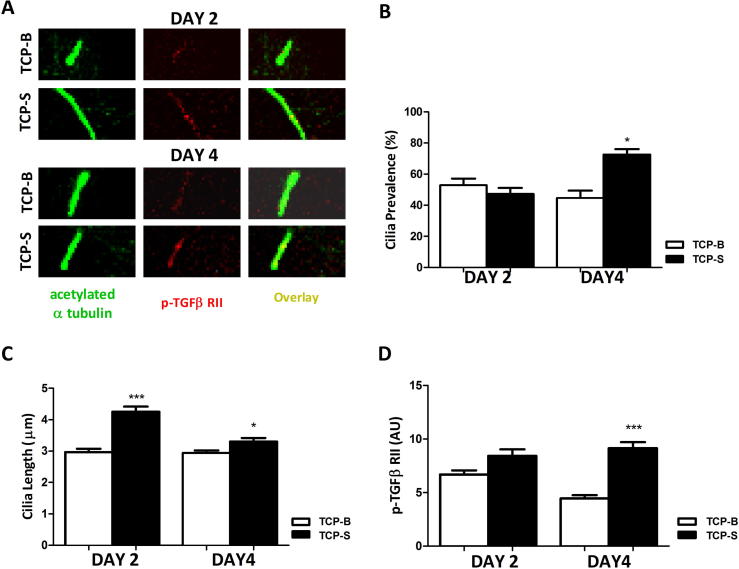
hBMSCs grown on TCP-S surfaces have longer cilia and increased ciliary p-TGFβ RII over four days of culture. (A) Representative images of primary cilia labelled for acetylated α-tubulin (left, green) of hBMSCs cultured on TCP-B and TCP-S surfaces at day 2 and day 4 of culture with corresponding images labelled for p-TGFβ RII (middle, red). Overlaid images were shown at right. Scale bars 2 µm. (B) Cilia prevalence, (C) length and (D) mean ciliary p-TGFβ RII intensity for each group as described above for day 2 and day 4. n = 100–110 cilia measured per group for (C) and (D), n ≥ 5 fields per group with ≥15 cells/field for (B). ^*^: p < 0.05, ^***^: p < 0.001, TCP-S against TCP-B at correlating time point. (For interpretation of the references to colour in this figure legend, the reader is referred to the web version of this article.)

Ciliary length and p-TGFβ RII levels were investigated at day 2 and day 4 of culture by confocal immunofluorescent imaging of acetylated alpha tubulin and p-TGFβ RII (Fig.4A). By day 4, cilia prevalence was significantly greater on TCP-S ceramics compared to TCP-B (Fig.4B) reflecting the difference observed at day 7 (Fig.3B). Culturing cells on TCP-S caused cilia elongation at both day 2 and day 4 compared to cells on TCP-B, with greatest cilia length observed at day 2 (Fig.4C). Localisation of p-TGFβ RII on the cilia axoneme was higher in hBMSCs grown on TCP-S than on TCP-B at both day 2 and day 4, the difference being statistically significant by day 4 (Fig.4D, p < 0.001).

### 3.5 Proliferation and osteogenic differentiation of hBMSCs are enhanced on TCP-S surfaces

**Fig. 5:**
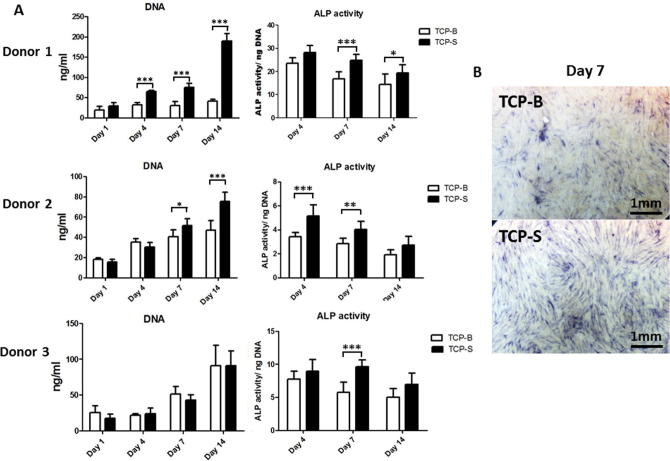
hBMSCs cultured on TCP-S show increased proliferation and osteogenic differentiation compared to cells on TCP-B. DNA quantification and ALP activity (normalized to DNA amount) of hBMSCs from 3 donors cultured on TCP discs over a 14-day period (A). Representative images of hBMSCs cultured for 7 days on TCP surfaces and showing more intense blue ALP staining on TCP-S (B). ^*^: p < 0.05, ^**^: p < 0.005, ^***^: p < 0.001, TCP-S against TCP-B at correlating time point. (For interpretation of the references to colour in this figure legend, the reader is referred to the web version of this article.)

Cells from 3 donors (n = 3) displayed increased proliferation over time from day 1 to day 14 on TCP-S, while cell proliferation on TCP-B varied with donors. Cells from one donor did not display any difference in proliferation rate on TCP-B over the time course studied. Enhanced cell proliferation on TCP-S was observed in hBMSCs from all the donors, two of them being significantly higher compared to the cells grown on TCP-B (Fig.5A, left panel).

ALP production varied among the donors (Fig.5A, right panel). In particular, ALP activity slightly decreased over time from day 4 to 14 in cells from donor 1 and 2 on both TCP ceramics, while those from donor 3 did not change their ALP activity with time from day 4 to 14. When TCP-S and TCP-B were compared, significantly higher ALP activity on TCP-S than on TCP-B was observed (Donor 1: at day 7 and 14; Donor 2: at day 7 and 14; donor 3: at day 7). ALP immuno-staining of a set of 7-day samples displayed increased ALP activity on TCP-S (Fig.5B).

### 3.6 Osteogenic gene expression is enhanced on TCP-S surfaces

**Fig. 6:**
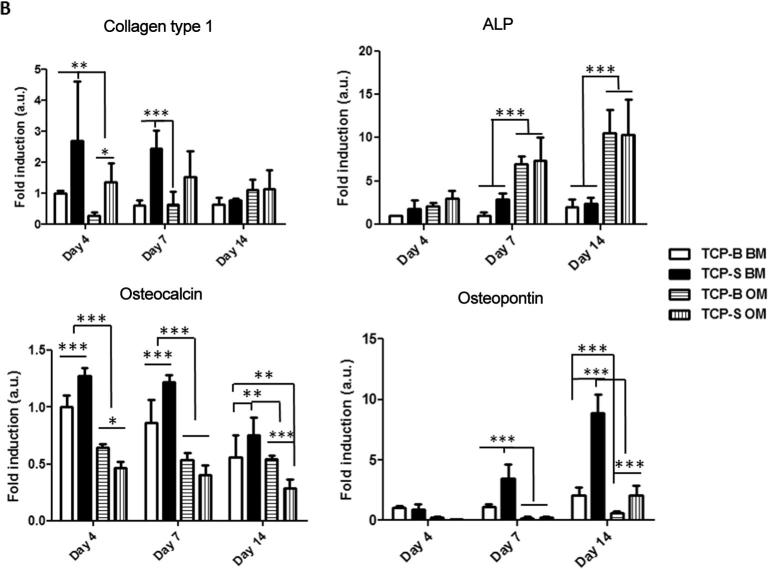
hBMSCs cultured on TCP-S show increased osteogenic gene expression compared to cells on TCP-B. Osteogenic genes expression profile of hBMSCs on TCP-S and TCP-B discs in both BM and OM normalized to the B2M (housekeeping gene). ^*^: p < 0.05, ^**^: p < 0.005, ^***^: p < 0.001.

Col I expression was downregulated in BM over time on TCP-S, while there was no significant change in hBMSCs cultured on TCP-B (Fig. 6). ALP gene expression was up-regulated in BM from day 4 to 7 and slightly down-regulated from day 7 to 14 on TCP-S. There was no significant difference of ALP gene expression on TCP-B in BM between day 4 and day 7, but it was slightly increased at day 14. Down regulation of OCN in BM was observed on both TCP ceramics from day 7 to day 14, but not significant. The most significant up-regulation in BM was observed for OPN on TCP-S, which was 4 times higher at day 7 and 10 times higher at day 14 compared to day 4, but only slightly up regulated (less than 2 times) from day 4 to day 14 on TCP-B. Comparing the cells on TCP-S and TCP-B, Col I expression in BM was significantly higher on TCP-S at day 4 and 7. ALP gene expression in BM was approximately three times higher on TCP-S at day 7 compared to TCP-B. OCN expression in BM was also enhanced on TCP-S at day 4, 7 and 14 compared to that observed on TCP-B. OPN expression displayed the most striking difference amongst osteogenic markers analyzed in BM on TCP-S and TCP-B. It was significantly increased on TCP-S, being three times higher at day 7 and five times higher at day 14 compared to that observed on TCP-B.

Comparing osteogenic gene expression of hBMSCs cultured on TCP discs in BM and OM revealed that dexamethasone in OM exerted a down-regulatory effect on Col I, OCN and OPN expression, but up-regulated ALP expression. In OM, TCP-S enhanced Col I gene expression at day 4 and 7, and OCN and OPN gene expressions at day 14. The enhancement of ALP gene expression on TCP-S in BM disappeared in OM.

### 3.7 TCP-S induces greater bone formation *in vivo*

**Fig. 7:**
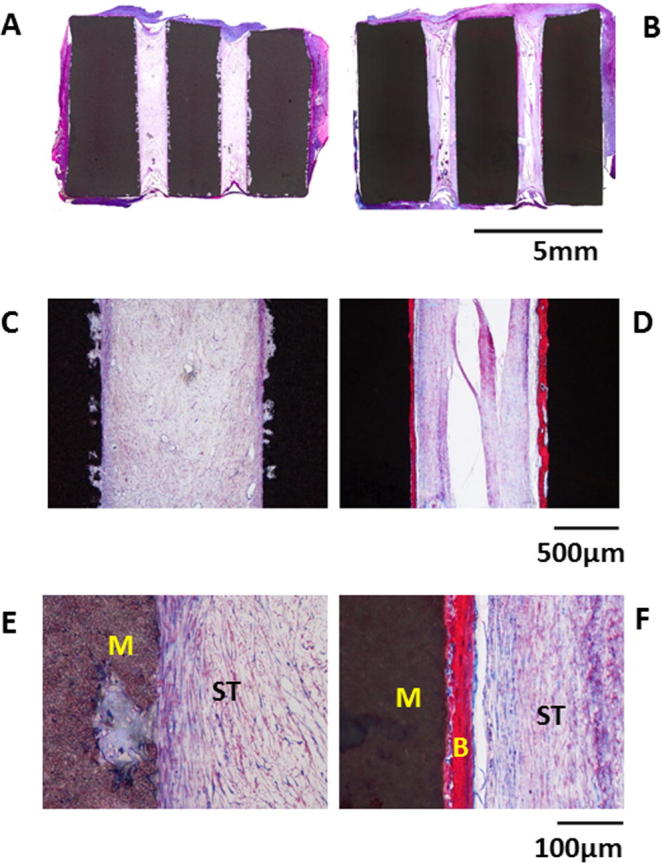
Inductive bone formation around TCP-S but not TCP-B in canine *in vivo* model. Histological overviews of TCP-B (A, C) and TCP-S (B, D) samples after a 12-week implantation in muscle of dog, showing bone formation in TCP-S and no bone in TCP-B; a high magnification image of TCP-B (E) explants, showing the infiltration of fibrous tissue but absence of bone in TCP-B; a high magnification image of TCP-S (F) explants, showing the presence of mineralized bone matrix (bright pink). (M: materials; B: bone; ST: soft tissue). (For interpretation of the references to colour in this figure legend, the reader is referred to the web version of this article.)

In total, 8 samples per TCP ceramic were harvested from 8 dogs for histological evaluation and histomorphometry. No bone formation was observed in any of the TCP-B samples (Fig.7A, C, E), while bone was formed in 7 out of 8 TCP-S explants (Fig.7B, D, F). Images with high magnification showed the presence of bone on the inner surface of TCP-S samples (Fig.7F) and only soft tissues were observed in TCP-B (Fig.7E). Quantitatively, 28 ± 17% of the available inner surface of TCP-S explants was occupied by bone.

## 4 Discussion

Chemical design of materials and application of biological molecules are often used to achieve specific biological responses in tissue regeneration. However there is an increasing amount of evidence suggesting that physical, mechanical or topographical properties of biomaterials also play a pivotal role in controlling biological functions [27]. In particular, it has been reported that the micro- and nano-structured surfaces of biomaterials can mediate cellular behavior including adhesion, morphology, proliferation and differentiation *in vitro* [28], [29]. Here, we reported that surface structure or topography of TCP ceramics affect hBMSC morphology, primary cilia expression and ciliary recruitment of p-TGFβR II *in vitro*. Furthermore, these differences were associated with regulation of osteogenesis. Thus submicron scaled surface features (TCP-S) induced greater cell spreading (Fig. 2), increased primary cilia expression, cilia elongation and recruitment of p-TGFβR II into the cilium (Fig. 3, Fig. 4) associated with increased osteogenic differentiation at both protein (Fig. 5) and gene level (Fig. 6). Furthermore, following an ectopic implantation, this material (TCP-S) also gave rise to heterotopic bone formation in muscle while TCP ceramic implants with micron scaled surface structure (TCP-B) did not (Fig. 7). These findings suggest for the first time, that the topographical cues may drive osteogenic differentiation by modulating primary cilia structure and ciliary recruitment of p-TGFβR II, which is required to activate TGFβ signalling.

The implantation of materials *in vivo* always follows the wound healing processes inherent of the innate immune reaction, followed by angiogenesis, tissue formation and remodeling. Many cell types (e.g. macrophages, myoblasts, MSCs and pericytes) involved in wound healing may be sensitive to surface structures and contribute to tissue morphogenesis. In the case of ectopic bone formation, osteogenic differentiation of MSCs infiltrated into the implants is crucial to induce bone regeneration [8]. A previous study has also demonstrated that BMSCs were involved in inductive bone formation in osteoinductive CaP ceramics [30]. Hence, our *in vitro* studies focused on the response of hBMSCs to the surface structure of TCP ceramics osteogenic differentiation.

The induction of stem cell osteogenesis by surface structure is dependent on the geometry and size of the surface structure, its spatial organization and the dynamical changes of the surface properties during time [31]. Col I, ALP, OCN and OPN genes are pivotal factors in osteogenic differentiation, matrix deposition and mineralization [13], [31], [32]. Without the presence of osteogenic factors, TCP-S not only promoted ALP activity but also up-regulated all bone-related gene expression tested in this study (Fig. 6). As the early osteogenic differentiation marker, ALP gene expression was slightly up-regulated by TCP-S at day 7 in BM (the maximal ALP gene expression in BM in the experimental time). With the presence of dexamethasone (osteogenic medium), ALP gene expression was largely up-regulated and increased with time up to day 14. This result was in line with the literature data showing the increase of ALP gene expression up to 14 days and the decrease thereafter in osteogenic medium [33]. No up-regulation of OCN over time from day 4 to day 14 was observed in this study, it might be possible that the enhanced gene expression of OCN could be expected at the late time points (e.g. day 21 or day 28) since OCN is a late osteogenic differentiation marker [33]. Given the fact that no growth factors were used, the gene expression in BM indicates that the dimension of surface structure alone could instruct osteogenic differentiation of hBMSCs. This result is in line with our previous data showing osteogenic regulation by the surface structure in 3D TCP ceramic granules [13].

The reaction of stem cells to the surface structure of the biomaterials is one of the first steps required for osteogenic differentiation induction [34]. The possibility of cells to make successful protrusions and contacts in a given direction varies with the size of the surface feature [35]. In other words, cells adapt their morphology according to the surface topography of the substrates they are cultured on [36]. It is known that cell differentiation is often associated with the morphological changes of the cells [17], [37]. For instance, when hBMSCs were cultured on substrates with adhesive islands of various sizes, changes in cell shape that occurred depending on the space available was responsible for hBMSC commitment to either adipogenic or osteogenic lineages [17], [38]. In particular, hBMSCs with rounded morphology differentiated into adipose cells, while hBMSCs with more spread morphology underwent osteogenic differentiation. The same correlation between cell morphology and osteogenic differentiation of hBMSCs was also seen in response to TCP ceramics in this study. Cells on both TCP-S and TCP-B were elongated, but those on TCP-S were more spread (Figs. 2) and underwent greater osteogenic differentiation (Fig. 5, Fig. 6).

Changes in cell morphology have been associated with alterations in intracellular cytoskeletal tension leading to altered expression of integrins and cadherins [17], [39] and primary cilia structure [40]. Recent findings show that cytoskeletal modulation by substrate topography affects primary cilia structure thereby regulating Wnt signalling in hBMSCs [20]. Furthermore primary cilia have been shown to be required for hBMSC osteogenic differentiation [21] and to undergo cilia elongation during osteogenesis as confirmed in the present study (Fig.3C) [26], [41]. These previous studies prompted the question as to whether the osteoinductive properties of TCP-S ceramics are associated with changes in primary cilia structure and function resulting from the alterations in cell morphology.

Previous reports have shown that TGFβ can induce osteogenic differentiation in hBMSCs [23]. In addition, TGFβ signalling is regulated by substrate stiffness and cytoskeletal tension, although the underlying mechanisms are unknown. Studies from Christensen’s group have shown that primary cilia regulate TGFβ signalling in fibroblasts associated with the recruitment of TGFβ receptors to the cilia axoneme [22]. Furthermore Hoey’s group have also shown how the receptors and downstream components in TGFβ signalling are localised to primary cilia in mesenchymal stem cells associated with activation of SMAD3 at the ciliary base [42]. In the present study, we observed that primary cilia length was increased on osteoinductive TCP-S ceramics and that this was associated with increased ciliary p-TGFβ RII. The time lag between initial cilia elongation and TGFβRII ciliary localisation may reflect slightly different intraflagellar transport (IFT) dynamics/mechanisms governing the movement of tubulin and TGF receptors onto the axoneme. The increased ciliary localization of TGF receptors, as seen on TCP-S ceramics, could provide a specialized environment to increase interactions between TGF pathway components hence causing increased TGFβ pathway activation that is essential in initiating osteogenic differentiation of hBMSCs. Our data therefore suggest for the first time, that surface topography regulates primary cilia structure and associated TGFβ RII localisation in the cilium, thereby enabling TGFβ signalling which is necessary for osteogenic differentiation of hBMSCs. However, it should also be noted whilst TGFβ signalling drives early osteogenesis, it is inhibitory at later stages. Interestingly we have shown previously that prolonged TGFβ causes primary cilia shortening [26], which may therefore act as a feedback mechanism down modulating TGFβ signalling and osteogenesis at later stages.

In addition to regulation of TGFβ signalling, primary cilia also regulate a number of other osteogenic signalling pathways, such as Wnt [20], [43], runx2 [44], and hedgehog [45], [46]. Indeed, previous studies from Knight’s group have shown that changes in primary cilia length can modulate both hedgehog and Wnt signalling pathways [20], [42], [44], [45]. Furthermore, in mesenchymal stem cells, primary cilia are necessary for mechanosignalling [47] which also regulated differentiation. Other recent studies report that cilia elongation increases mechanosignalling in bone [48]. Therefore changes in cilia length in response to osteogenic TCP topography may modulate *in vivo* bone formation through altered mechanosignalling in addition to regulation of osteogenesis.

Finally, dexamethasone is often used as an osteogenic factor, because it is essential for the full differentiation of hBMSCs into mineral-producing osteoblasts as shown by increased ALP activity [49], [50]. However, in the present study, hBMSCs cultured in OM with dexamethasone on TCP ceramics showed reduced levels of Col I, OCN, and OPN gene expression compared to cells in BM. These data suggested that ALP expression could be uncoupled from Col I, OCN and OPN, in agreement with previous studies showing that dexmethasone inhibited or postponed Col I and reduced OCN expression [51], [52]. Interestingly the surface structure of the osteoinductive TCP-S ceramics had a similar effect on primary cilia length with that induced by dexamethasone in the osteogenic media for cells cultured on glass (no difference in cilia length between OM Glass vs BM TCP-S, Fig.4C). This further highlights the potential potency of topography and cilia modulation as a means of controlling osteogenesis.

In addition to direct topography induced cilia elongation, it cannot be ruled out that changes in topography may influence the local surface chemistry and local ion release which may impact on osteogenesis as previously described [53]. It has been shown that an apatite layer was formed on the surface of TCP-S without detectable surface structure change when TCP-S was contacted to body fluids (e.g. culture medium) [13].

## 5 Conclusion

In this study we compared TCP ceramics with micron or sub-micron scaled surface structure, termed TCP-B and TCP-S respectively. TCP ceramic with a submicron scale surface induced a more spread stem cell morphology, increased expression and length of primary cilia, recruitment of p-TGFβ RII to the ciliary axoneme and osteogenic differentiation at a cellular and molecular level without any additional osteogenic factors *in vitro*. Furthermore, this osteogenic response was associated with increased inductive bone formation *in vivo*. These data not only highlight the importance of topography in regulating osteogenesis but also imply a novel mechanism involving primary cilia elongation and recruitment of p-TGFβ RII to the ciliary axoneme. This may therefore represent a new biomaterial based ‘*ciliotherapy*’ for use in orthopedic, dental and maxillofacial surgery applications.

## Contributions

All primary cilia and TGFβ experimental work was conducted by MD with support from PC and MK at Queen Mary University of London. The preparation and characterization of TCP, the *in vitro* analysis of ALP and osteogenic gene expression was conducted by JZ with support from XL, EV, DB, LM and HY at Maastricht University. *In vivo* analysis was conducted by HY at Sichuan University. JB, CB provided ideas. JZ, MD, MK, and HY conceived the study and wrote the paper. All authors were involved in analysis of different aspects of the results.
